# The impact of changes in opioid dependency treatment upon COVID-19 transmission in Sydney, Australia: a retrospective longitudinal observational study

**DOI:** 10.1186/s12889-024-17827-0

**Published:** 2024-02-02

**Authors:** Benjamin T. Trevitt, Victoria Hayes, Rachel Deacon, Llewellyn Mills, Apo Demirkol, Nicholas Lintzeris

**Affiliations:** 1 Drug and Alcohol Services, South Eastern Sydney Local Health District, Sydney, NSW Australia; 2 School of Public Health and Community Medicine, UNSW Sydney, Sydney, Australia; 3 Speciality Addiction Medicine, Faculty Medicine and Health, University of Sydney, Sydney, NSW Australia; 4 Drug and Alcohol Clinical Research and Improvement Network, Sydney, NSW Australia

**Keywords:** COVID-19, Opioid dependency treatment, Takeaway dose, Vaccination, Methadone, Sublingual buprenorphine, Long acting depot buprenorphine, Harm reduction, Vulnerable populations

## Abstract

**Background:**

In April 2020, in response to the COVID-19 public health emergency, South Eastern Sydney Local Health District (SESLHD) Drug and Alcohol services modified their delivery of opioid dependency treatment (ODT) to reduce spread of COVID-19 and maintain continuity of care by increasing use of takeaway doses (TADs), transferring clients to local community pharmacies for dosing and encouraging the use of long-acting depot buprenorphine (LADB) which enabled once a month dosing.

**Methods:**

This study was a retrospective longitudinal case–control study conducted from August 1st, to November 30th, 2021. Eligible clients were those admitted for treatment with SESLHD ODT Services prior to August 1st,2021 and who remained in treatment beyond November 30th, 2021. COVID-19 diagnoses were determined by a COVID-19 PCR and extracted from the electronic Medical Records (eMR) Discern Reporting Portal. Demographic, clinical and dosing related data were collected from eMR and the Australian Immunisation Register (AIR).

**Results:**

Clients attending SESLHD ODT services had significantly greater odds of acquiring COVID-19 than the NSW adult population at large (OR: 13.63, 95%CI: 9.64,18.88). Additionally, amongst SESLHD ODT clients, being of Aboriginal and Torres Strait Islander origin was associated with greater odds of acquiring COVID-19 (OR = 2.18, CI: 1.05,4.53); whilst being employed (OR = 0.06, CI:0.01,0.46), receiving doses at pharmacy (OR = 0.43, CI: 0.21,0.89), and being vaccinated (OR = 0.12, CI: 0.06,0.26) were associated with lower odds. Every additional day of attendance required for dosing was associated with a 5% increase in odds of acquiring COVID-19 (OR = 1.05, CI: 1.02,1.08).

**Conclusions:**

Clients attending SESLHD ODT services are significantly more likely to acquire COVID-19 than the NSW population at large. Promoting vaccination uptake, transferring clients to pharmacy, and reducing the frequency of dosing (by use of takeaway doses or long-acting depot buprenorphine) are all potential methods to reduce this risk.

## Background

Coronavirus (COVID-19) is an infectious disease caused by severe acute respiratory syndrome coronavirus 2 (SARS-CoV-2) [1]. It was first detected in Wuhan City, China, in December 2019 during a local outbreak of respiratory illnesses [1] and by March 2020 the World Health Organisation had declared it a global pandemic [2]. At the time of writing, Australia had already been exposed to eight different waves of COVID-19 [3], however the delta-wave outbreak (June 16, 2021 to December 15, 2021) was one of the most fatal, especially for younger populations [3]. Over eighty thousand COVID-19 cases were recorded in New South Wales (NSW) during this timeframe (mean = 453.4 cases/day), with a case fatality rate of 0.71% [3]. Although extensive public health measures had already been implemented to reduce case numbers and fatalities at the time of the delta wave outbreak, (e.g. legislation enforcing mask wearing and isolation of cases, and campaigns promoting vaccination uptake) [1, 2], the highly contagious and evolving nature of the virus meant that case fatality rates still inevitably remained high. Moreover, beyond case-fatalities, there is now a growing body of evidence that COVID-19 has long-term psychological (e.g. anxiety and depression) and physical (e.g. palpitations, sleep difficulty, hair-loss, myalgia, fatigue, anosmia and dizziness) impacts on the quality of life of many of its survivors [4, 5]. It has also profoundly impacted the health of the general population, particularly health-care workers, due to increased work-related stressors [6].

People with substance use disorders (SUDs) have higher rates of comorbidities, (such as mental health, diabetes, chronic obstructive pulmonary disease, chronic kidney disease and hepatitis C) [7, 8], than the general population. Additionally, they often face significant social disadvantages, such as homelessness, poverty, and discrimination, which can be barriers to healthcare service access [7, 9, 10]. Consequently, not only are they more vulnerable to a more severe COVID-19 illness but they are also less likely to have access to the support, information, and tools to facilitate and/or enable COVID-19 prevention, screening, and treatment [7, 9]. Emerging data, largely from North America, has illustrated that people with SUDs are significantly more likely to develop [8, 10–13], be hospitalised [8, 10–13] or die [8, 10–13] from COVID-19 than people without SUDs. Indeed, one large retrospective case control study (*n* = 73,099,850) conducted in North America in 2020 [8] found that, of all substance users, those with opioid use disorders (both recent and long-term diagnosis) were the most at risk of COVID-19 acquisition compared to non-substance users (OR = 10.2 (9.1–11.5) and 2.4 (2.2–2.6)) respectively) [8]. Thus, urgent implementation of public health measures targeting this at-risk group was critical.

South Eastern Sydney Local Health District (SESLHD) Drug and Alcohol Services have provided a range of inpatient and community-based treatment and harm reduction services for people with SUDs for over 60 years [14]. This includes provision of opioid dependence treatment (ODT) with either oral methadone, sublingual buprenorphine (SL-BPN) or long acting depot buprenorphine (LADB) to between 520 to 600 clients at any one time, as well as counselling and social support [14]. Prior to COVID-19, ODT had conditions requiring daily attendance for dosing at either clinics or community pharmacies (for methadone and SL-BPN), with limited takeaway doses (TADs) available for clients assessed as ‘low risk’ for safety [15]. COVID-19 raised significant concerns for ODT services in their ability to implement effective social distancing, personal protective equipment (PPE) and isolation of cases- necessary to protect clients, staff, and their families [7, 15–17]. Additionally, according to some recent international studies, clients with SUDs had (and still have) significantly lower vaccination rates than the rest of their communities [18–23].

In April 2020, in response to the public health emergency, SESLHD dramatically modified its delivery of ODT with the aims of reducing the spread of COVID-19 and maintaining continuity of care [15, 16] by: increasing use of TADs; transferring clients to local community pharmacies for dosing; encouraging the use of LADB (enabling once a month dosing); increasing the use of telehealth services with clients; introducing perimeter screening of staff and clients entering clinic settings; enforcing the use of PPE for staff and mask wearing for clients in clinical settings; enforcing mandatory COVID-19 vaccination of staff; encouraging and providing COVID-19 vaccination to clients; and ensuring access to reverse transcriptase polymerase chain reaction (PCR) (and later rapid antigen [RAT]) testing for staff and clients [15, 16]. Other societal changes occurred in Australia outside of the treatment setting, at various periods of the COVID-19 pandemic, including provision of emergency housing for people with unstable accommodation, greater economic assistance for people with reduced employment [24, 25], restrictions to travel in the community [26], and free COVID-19 vaccination rollouts via medical practices and pharmacies [26].

Many evaluations of ODT service changes in response to COVID-19 have demonstrated that neither increasing the number of TADs of methadone or SL-BPN, nor switching clients to LADB treatment was associated with negative treatment outcomes or poorer treatment adherence [15, 27–31]. Additionally, there is evidence that clients who received more TADs or who were on LADB had better continuity of care when in isolation, higher treatment retention, no significant increase in substance use, and a higher satisfaction with treatment and care in general [15, 31].

However, we believe that this is the first study to evaluate whether these service changes were effective in reducing the spread of COVID-19 amongst ODT clients. This study will focus on the delta COVID-19 wave which commenced in NSW mid-2021 [32] and specifically the 4-month period August 1st, 2021 to November 30th, 2021. The reason behind the above timeframe is the relatively low total number of COVID-19 cases that had occurred across SESLHD (a population catchment of 1 million people) and the whole of NSW (population of 8.2 million) [33] prior to August 2021 (*n* = 928 and *n* = 8,725 respectively) [34–36] compared to the significantly larger number that occurred between August 1st, and November 30th, 2021 (*n* = 2,366 and *n* = 73,002 respectively) [34–36]. In fact, SESLHD ODT services had record of only one COVID-19 positive case occurring prior to June 2021. Additionally, during the selected timeframe, only PCR tests were considered diagnostic [37], and all positive PCR tests were automatically recorded centrally in a large database, the NSW Notifiable Conditions Information Management System (NCIMS) [38]. However, in November 2021, due to the increasing number of positive cases, the Therapeutic Goods Administration began authorising the sale of select self-test rapid antigen test (RAT) kits [39]. RAT tests were required to have a clinical sensitivity of at least 80% and a clinical specificity of at least 98% [40, 41] and by January 2022 were considered sufficient for a positive COVID-19 diagnosis in NSW [37, 42]. This meant accurate capture of all positive cases became increasingly difficult as positive results were not automatically reported to NSW Health.

The primary aim of this study was to determine whether treatment characteristics (dosing site, frequency required to attend clinic/pharmacy for dosing and medication type [methadone, SL-BPN, LADB]) or client characteristics (demographics, housing, employment, and vaccination status) significantly impacted on COVID-19 infection rates in ODT populations. The secondary aim was to identify whether infection rates of SESLHD ODT clients were comparable with the infection rates of NSW at large.

## Methods

This study employed a retrospective longitudinal case control design of ODT clients who attended SESLHD Drug and Alcohol Services from August 1st, to November 30th, 2021. It examined the impact of location and type of treatment, frequency of attendance, and client characteristics, on COVID-19 infection rates. To be eligible for inclusion, ODT clients had to be admitted for treatment with SESLHD Drug and Alcohol Services prior to August 1st, 2021 and have remained in treatment beyond November 30th, 2021. Clinical data was sourced from the electronic medical record (eMR) system which is routinely used in NSW healthcare settings [43] and cross-checked, where necessary, with iDose (an electronic medication device which records doses of methadone, SL-BPN and LADB administered at clinics) [44].

### Drug and alcohol services for ODT

SESLHD Drug and Alcohol Services provides ODT to approximately 520 to 600 clients at any one time [15, 45] within multidisciplinary teams (consisting of doctors, nurses, allied health, pharmacists and consumer workers) [15]. All services are free for clients, except for private dispensing fees for those dosing at community pharmacies. As of August 2021, 49% of SESLHD ODT clients were on methadone, 16% on SL-BPN and 35% on LADB. Dosing of methadone and SL-BPN could occur at either clinic or pharmacies with TADs available at pharmacies only, whilst LADB dosing was only available at clinics [15]. Further information regarding the change in location, type of dosing, and number of TADs administered to clients in response to the COVID-19 pandemic is available in a previous research paper [15].

### Study team and participants

The study team consisted of clinicians, consumer-workers and researchers from SESLHD Drug and Alcohol Services who contributed to the development of the key research questions, study design, data-analysis and write-up. The participants were clients who received ODT services through SESLHD for the entire four-month period of August 1st, to November 30th, 2021. Client demographics and ODT treatment conditions were captured in SESLHD electronic medical records (eMR) [43]. All clients who tested positive for COVID-19 during this time frame were extracted from the Discern Reporting Portal, a feature of eMR which allows one to run various reports and extract information such as COVID-19 diagnoses [43]. All information recorded was de-identified. This study was retrospectively conducted by SESLHD healthcare workers with input from the Aboriginal Health Unit to ensure it was handled in a culturally appropriate manner. Research was performed in accordance with the guidelines and regulations presented in the Declaration of Helsinki. The SESLHD Human Research Ethics Committee (HREC),^1^ at a meeting of its Low and Negligible Risk Research Review Committee, waivered the need for informed consent and ethical approval for this study. It also determined that the study did not raise any ethical risks requiring submission to an ethical review committee in accordance with NSW Health Policy.

### Evaluation period, eligibility, data sources and measures

All clients enrolled in ODT with SESLHD Drug and Alcohol Services for the entire time-period were eligible for inclusion in the study. Data from the Australian Treatment Outcome Profile (ATOP) questionnaires [46], eMR entries [43], patient paper-files, and iDose [44] between August 1st, 2021 and November 30th, 2021 were captured to determine basic demographics (gender, age, housing situation, and employment status) and details of current opioid treatment (medication type, TADs, and dosing site). Data were exported from the Discern Reporting Portal in eMR [43] and the Australian Immunisation Register (AIR) [47] to determine case and vaccination status of clients respectively.

### Client descriptors

Client descriptors including demographics, substance use and social conditions were measured by ATOP—a brief questionnaire which has been validated in Australian Drug and Alcohol treatment populations [15, 46]. The ATOP includes client-reported information regarding recent substance use (number of days out of the previous 28 that they used substances), ratings of physical health, psychological health and overall quality of life on scales from one (poor) to ten (excellent), about recent occupational status (proportion of the last 28 days spent working/studying) and are assessed for homelessness risk and recent experience of violence [15, 46]. ATOPs are routinely completed every two to three months as part of routine clinical care [15, 46].

### Treatment descriptors

The *Opioid Substitution Treatment Module* [15] details a client’s prescribed medication, (methadone, SL-BPN or LADB), dosing location, and the number of TADs received per week. This module is routinely completed by the prescribing doctor every 2-to-3 months, or following any change in medication conditions.

*iDose* [44] details the frequency of attendance, ODT medication and dose of clients who attend SESLHD ODT clinics for dosing.

### COVID-19 and Vaccination status measurements

A COVID-19 diagnosis was determined from a COVID PCR as it was considered the gold-standard at the time [48]. Positive COVID-19 diagnoses were extracted from the eMR Discern Reporting Portal [43] after being uploaded by the SESLHD Public Health Unit (PHU).

A fully vaccinated status was defined as having received at least two doses of approved COVID-19 vaccines, as this was the NSW health criteria at the time [49]. A client’s vaccination status was extracted from the AIR [47].

### Data analysis

Data was exported from eMR [43], the AIR [47] and iDOSE [44] to Microsoft Excel where it was cleaned and collated. Data analysis was conducted using STATA v17.

Descriptive analyses of clients’ demographics (age, gender and Aboriginal and Torres Strait Islander^2^ status, and employment, housing, and vaccination statuses) were undertaken and compared based on ODT characteristics (e.g. medication type, dosing location and frequency of attendance required for dosing). Differences between groups were determined by comparing means and calculating odds ratios using linear and logistic regression models respectively.

Univariate and multivariate logistic regression models were developed using demographic variables (i.e. age, gender, employment and housing status) and treatment conditions (i.e. dosing location, attendance requirements and medication type) to assess which demographic and treatment characteristics were significantly protective against COVID-19 infection. A new covariate ‘number of days the client was required to attend per month for dosing’ was created to replace the covariate ‘number of takeaways per week’, to allow for inclusion of clients receiving LADB in the analysis.

To determine which covariates to include in the final multivariate model, data was split into training and validation subsamples and the least absolute shrinkage and selection operator (lasso) was utilised to estimate model coefficients. Cross-validation, adaptive lasso and plug-in methods of tuning parameter estimation were compared based on out-of-sample mean squared error (MSE).

## Results

### Participants

**Fig. 1:**
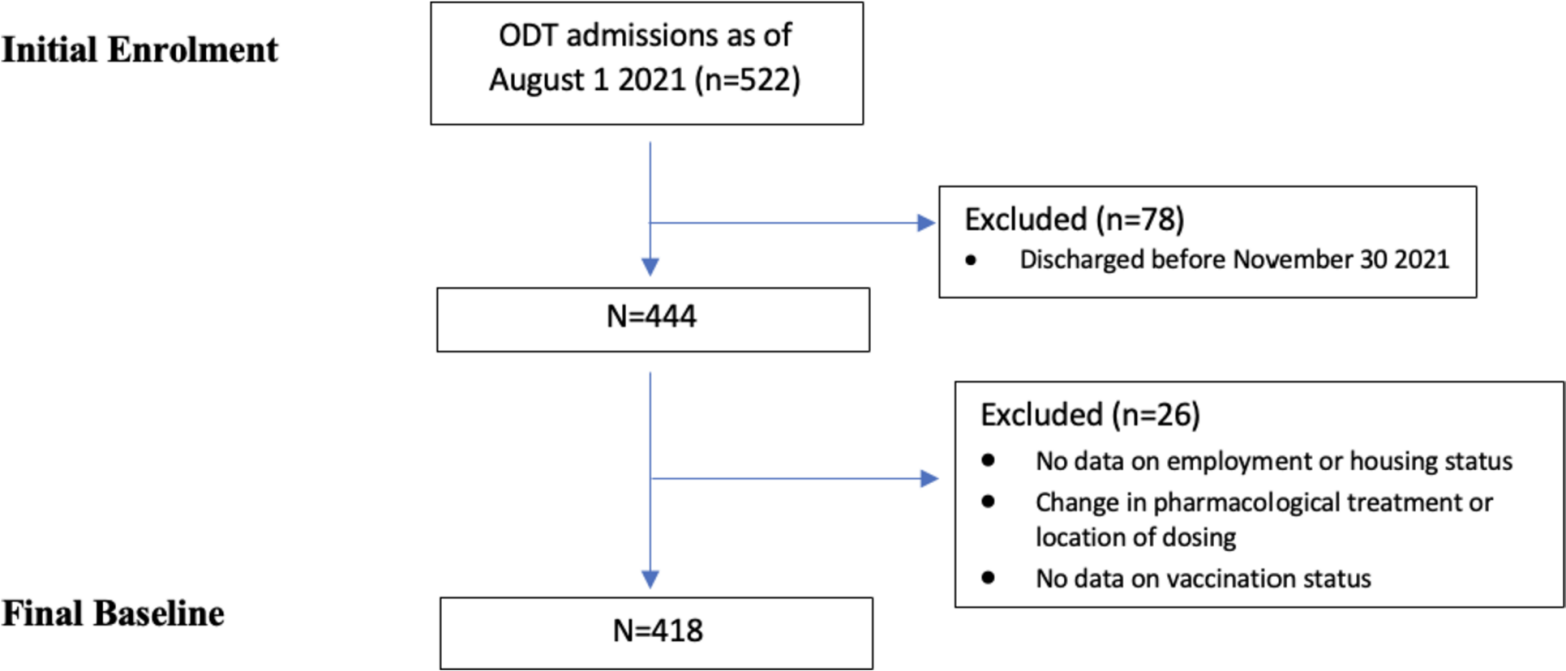
Flow chart of eligibility requirements for inclusion in the study

As of August 1st, 2021 there were 522 clients enrolled in treatment at SESLHD ODT Drug and Alcohol Services. Clients discharged from the service prior to November 30th, 2021 (*n* = 78) were excluded from further analysis. Of the 444 remaining clients four had no recorded information about employment or housing status, fourteen changed pharmacological treatment or location of dosing during the above time frames, and eight had no data on vaccination status. 418 (94%) of eligible clients had complete data and were thereby included in baseline analysis (Fig. 1).

Demographics and baseline characteristics of the 418 ODT clients were compared based on prescribed opioid (Table 1), place of dosing (Table 2), and number of TADs (Table 3).

**Table 1:** Characteristics of clients receiving ODT by prescribed opioid

**Characteristic**	**Methadone (M) (*n* = 202)**	**SL-BPN (S) (*n* = 69)**	**LADB (L) (*n* = 147)**	**Total (*n* = 418)**	**Coefficient**	**Comparison estimate (95% CI)**^a^	***p*-value**
**Age**, numeric, in y, M (SD)	43.0 (10.15)	40.3 (12.9)	40.8 (11.01)	41.8 (11.00)	Beta	M-S: 2.7 (-0.31,5.69)	0.08
						M-L: 2.3 (-0.07,4.60)	0.06
						S-L: -0.4 (-3.57,2.71)	0.79
**Gender**, binary, (female [ref] vs male), n(%) male	146 (72%)	44 (64%)	89 (61%)	279 (67%)	Odds Ratio	M-S: 1.5 (0.83,2.64)	0.18
						**M-L: 1.7 (1.08,2.67) **	**0.02**
						S-L: 1.1 (0.63,2.07)	0.65
**Aboriginal client,** binary, (Non-Aboriginal [ref] vs Aboriginal), n(%) Aboriginal	42 (21%)	12 (17%)	20 (14%)	74 (18%)	Odds Ratio	M-S: 1.2 (0.61,2.53)	0.54
						M-L: 1.7 (0.93,2.98)	0.09
						S-L: 1.3 (0.61,2.92)	0.47
**Employment status,** binary, (Unemployed [ref] vs Employed), n(%) employed	40 (20%)	30 (43%)	41 (28%)	111 (27%)	Odds Ratio	**M-S: 0.3 (0.18,0.58)**	** < 0.001**
						M-L: 0.6 (0.39,1.05)	0.08
						**S-L: 2.0 (1.09,3.61) **	**0.02**
**Housing status** binary, (Not homeless [ref] vs Homeless), n(%) Homeless	8 (4%)	1 (1%)	10 (7%)	19 (5%)	Odds Ratio	M-S: 2.8 (0.34,22.83)	0.34
						M-L: 0.6 (0.22,1.47)	0.24
						S-L: 0.2 (0.03,1.61)	0.13
**Vaccination status** binary, (Not fully vaccinated [ref] vs Vaccinated), n(%) Vaccinated	135 (67%)	44 (64%)	107 (73%)	286 (68%)	Odds Ratio	M-S: 1.1 (0.65,2.03)	0.64
						M-L: 0.8 (0.47,1.20)	0.23
						S-L: 0.7 (0.36,1.21)	0.18

^a^Unadjusted estimates of beta coefficients and odds ratios have been presented in this table

**Table 2:** Characteristics of clients receiving ODT by Dosing Site

Characteristic	Pharmacy (P)	Clinic (C)	Total	Coefficient	Comparisons estimate (95% CI)^a^	*p*-value
**Age**, numeric, in y, M (SD)	42.2 (11.22)	41.5 (10.80)	41.8 (10.99)	Beta	P–C: 0.7 (-1.46,2.79)	0.54
**Gender**, binary, (female [ref] vs male), n(%) male	126 (67%)	153 (67%)	279 (67%)	Odds Ratio	P–C: 1.0 (0.68,1.54)	0.91
**Aboriginal client,** binary, (Non-Aboriginal [ref] vs Aboriginal), n(%) Aboriginal	33 (18%)	41 (18%)	74 (18%)	Odds Ratio	P–C: 1.0 (0.59,1.63)	0.94
**Employment status,** binary, (Unemployed [ref] vs Employed), n(%) employed	64 (34%)	47 (20%)	111 (27%)	Odds Ratio	**P–C: 2.0 (1.29,3.12)**	**0.002**
**Housing status** binary, (Not homeless [ref] vs Homeless), n(%) Homeless	2 (1%)	17 (7%)	21 (5%)	Odds Ratio	**P–C: 0.1 (0.03,0.59)**	**0.008**
**Vaccination status** binary, (Not fully vaccinated [ref] vs Vaccinated), n(%) Vaccinated	126 (67%)	160 (70%)	286 (68%)	Odds Ratio	P–C: 0.9 (0.59,1.35)	0.58

^a^Unadjusted estimates of beta coefficients and odds ratios have been presented in this table

**Table 3:** Characteristics of clients receiving ODT by weekly dosing site attendance

Characteristic	< = 1 time (A) (*n* = 235)	2–5 times (B) (*n* = 78)	6–7 times (C) (*n* = 105)	Coefficient	Comparisons estimate (95% CI)^a^	*p*-value
**Age**, numeric, in y, M (SD)	41.4 (11.57)	41.3 (9.91)	43.1 (10.37)	Beta	C-B: 1.8 (-1.43,5.02)	0.28
					C-A: 1.8 (-0.77,4.30) B-A: 0.0 (-2.85,2.79)	0.17 0.98
**Gender**, binary, (female [ref] vs male), n(%) male	141 (60%)	54 (69%)	84 (80%)	Odds Ratio	C-B: 1.8 (0.90,3.50)	0.10
					**C-A: 2.7 (1.55,4.60)**	** < 0.001**
					B-A: 1.5 (0.87,2.59)	0.15
**Aboriginal client,** binary, (Non-Aboriginal [ref] vs Aboriginal), n(%) Aboriginal	31 (13%)	18 (23%)	25 (24%)	Odds Ratio	C-B: 1.0 (0.52,2.08)	0.91
					**C-A: 2.1 (1.14,3.70)**	**0.02**
					**B-A: 2.0 (1.03,3.77)**	**0.04**
**Employment status,** binary, (Unemployed [ref] vs Employed), n(%) employed	80 (34%)	26 (33%)	5 (5%)	Odds Ratio	**C-B: 0.1 (0.04,0.28)**	** < 0.001**
					**C-A: 0.1 (0.04,0.25)**	** < 0.001**
					B-A: 1.0 (0.56,1.67)	0.91
**Housing status** binary, (Not homeless [ref] vs Homeless), n(%) Homeless	10 (4%)	1 (1%)	8 (8%)	Odds Ratio	C-B: 6.4 (0.78,51.88)	0.09
					C-A: 1.9 (0.72,4.84)	0.21
					B-A: 0.3 (0.04,2.32)	0.24
**Vaccination status** binary, (Not fully vaccinated [ref] vs Vaccinated), n(%) Vaccinated	172 (73%)	52 (67%)	62 (59%)	Odds Ratio	C-B: 0.7 (0.39,1.33)	0.29
					**C-A: 0.5 (0.33,0.86)**	**0.01**
					B-A: 0.7 (0.42,1.27)	0.27

Clients on methadone, SL-BPN and LADB did not differ significantly in terms of age. The odds of clients on methadone being male were significantly greater than that of clients on LADB. The odds of clients on methadone being employed were significantly less than that of clients on LADB or SL-BPN (Table 1).

The odds of clients dosing at pharmacy being employed were significantly higher than that of clients dosing at clinics. The odds of clients dosing at pharmacy being homeless were significantly lower than clients dosing at clinics (Table 2).

The odds of clients required to attend clinic or pharmacy six for more times per week for dosing (accessing methadone or SL-BPN) being male or identifying as Aboriginal were significantly higher than that of clients required to attend one or less times per week (accessing methadone or SL BPN TADs or in LADB treatment). The odds of clients required to attend clinic or pharmacy six or seven times per week for dosing being employed or vaccinated were significantly lower than that of clients required to attend one or less times per week (Table 3).

### Vaccination status

By the end of October 2021 *n* = 286 (68%) of SESLHD ODT clients had been double vaccinated. This was considerably lower than NSW, as a whole, which reported a double vaccination rate of 83% at that time [49].

Double vaccinated clients were required to attend dosing sites significantly less frequently than unvaccinated clients (Table 4). The odds of double vaccinated clients being employed were significantly higher than that of unvaccinated clients, whilst the odds of double vaccinated clients identifying as Aboriginal were significantly lower than that of unvaccinated clients.

**Table 4:** Characteristics of clients receiving ODT by vaccination status

Characteristic	Vaccinated (V) (*N* = 286)	Unvaccinated (U) (*N* = 132)	Coefficient	Comparisons estimate (95% CI)^a^	*p*-value
**Age**, numeric, in y, M (SD)	42.4 (11.14)	40.4 (10.56)	Beta	V-U: 2.0 (-0.24,4.30)	0.08
**Gender**, binary, (female [ref] vs male), n(%) male	191 (67%)	88 (67%)	Odds Ratio	V-U: 1.0 (0.65,1.56)	0.98
**Aboriginal client,** binary, (Non-Aboriginal [ref] vs Aboriginal), n(%) Aboriginal	43 (15%)	31 (23%)	Odds Ratio	**V-U: 0.6 (0.34,0.97)**	**0.04**
**Employment status,** binary, (Unemployed [ref] vs Employed), n(%) employed	91 (32%)	20 (15%)	Odds Ratio	**V-U: 2.6 (1.53,4.47)**	** < 0.001**
**Housing status** binary, (Not homeless [ref] vs Homeless), n(%) Homeless	13 (5%)	6 (5%)	Odds Ratio	V-U: 1.0 (0.37,2.69)	1.00
**Dosing location** binary, (Clinic [ref] vs Pharmacy), n(%) Pharmacy	126 (44%)	62 (47%)	Odds Ratio	V-U: 0.9 (0.59,1.35)	0.58
**Dosing Type** binary, (injection [ref] vs oral/SL), n(%) oral/SL	179 (63%)	92 (70%)	Odds Ratio	V-U: 0.7 (0.47,1.13)	0.16
**Monthly attendance for dosing, numeric, in days (M, SD)**	9.3 (10.60)	12.3 (11.54)	Beta	**V-U: -3.0 (-5.29,-0.77)**	**0.009**

^a^Unadjusted estimates of beta coefficients and odds ratios have been presented in this table

### Patient outcomes – COVID-19 univariate analysis

Univariate analysis revealed that the odds of Aboriginal clients acquiring COVID-19 during the study timeframe were significantly higher than that of non-Aboriginal clients; whilst the odds of employed clients and clients dosing at pharmacies acquiring COVID-19 were significantly lower than that of unemployed clients and clients dosing at clinics respectively (Table 5). Additionally, the odds of clients required to attend clinic or pharmacy one or less or between two and five times per week acquiring COVID-19 were significantly lower than that of clients required to attend six to seven times per week. These statistically significant results still held when clients receiving LADB and clients receiving oral ODT were analysed separately. In fact, every additional day per month of required attendance for dosing was associated with a 5% increase in odds of acquiring COVID-19.

**Table 5:** Univariate modelling and frequencies by covariates with COVID-19 acquisition as outcome

Characteristic	COVID-19 positive	OR (95% CI)	*p* value
**Age**, numeric, mean (SD), in y	41.8 (10.99)	1.01 (0.98,1.04)	0.58
**Gender**, binary, (female [ref] vs male)			
Female (n, %)	14 (10.1)		
Male (n, %)	26 (9.3)	0.92 (0.46,1.82)	0.81
**Aboriginal client,** binary, (Non-Aboriginal [ref] vs Aboriginal)		2.18 (1.05,4.53)	**0.04**
Non-Aboriginal (n, %)	28 (8.1)		
Aboriginal (n, %)	12 (16.2)		
**Employment status,** binary, (Unemployed [ref] vs Employed)		0.06 (0.01,0.46)	**0.007**
Unemployed (n, %)	39 (12.7)		
Employed (n, %)	1 (0.9)		
**Housing status** binary, (Not homeless [ref] vs Homeless)		1.83 (0.51, 6.59)	0.35
Not homeless (n, %)	37 (9.3)		
Homeless (n, %)	3 (15.8)		
**Dosing location** binary, (Clinic [ref] vs Pharmacy)		0.43 (0.21, 0.89)	**0.03**
Clinic (n, %)	29 (12.6)		
Pharmacy (n, %)	11 (5.9)		
**Dosing Type** binary, (Injection [ref] vs Oral)		1.30 (0.64,2.63)	0.47
Injection (n, %)	12 (8.2)		
Oral (n, %)	28 (10.3)		
**Vaccination status** binary, (not vaccinated [ref] vs fully vaccinated)		0.12 (0.06,0.26)	** < 0.001**
Not vaccinated (n, %)	30 (22.7)		
Fully vaccinated (n, %)	10 (3.5)		
**Weekly attendance rate,** (6 + [ref] vs 0–1, 2–5)			
0-1 times per week (overall)	15 (6.4)	0.29 (0.14,0.60)	**0.001**
0–1 times per week (inj. only)	5 (6.4)	0.38 (0.18,0.81)	**0.01**
0–1 times per week (oral only)	20 (19.5)	0.15 (0.04,0.52)	**0.003**
2–5 times per week		0.29 (0.10,0.81)	**0.02**
6 + times per week		Reference group	Reference group
**Number of days attending clinic/pharmacy for dosing, mean (SD)**	15.8 (12.4)	1.05 (1.02,1.08)	**0.001**

### Patient outcomes – COVID-19 multivariate analysis

The final multivariate model used as predictor for the likelihood COVID-19 acquisition was obtained using the cross-validation based lasso method and included covariates of vaccination status, dosing location, employment status, required attendance per month (in days), whether the client identified as Aboriginal, and age (Table 6). Covariates sex and homelessness were also included in the model, but their coefficients were reduced to zero.

**Table 6:** Multivariate modelling using the cross-validation based lasso method with COVID acquisition as outcome

Characteristic	OR (95% CI)	*p* value
**Employment status,** binary, (Unemployed [ref] vs Employed)	0.12 (0.01,1.00)	**0.05**
**Vaccination status,** binary, (Unvaccinated [ref] vs Vaccinated)	0.13 (0.06,0.29)	** < 0.001**
**Dosing location** binary, (Clinic [ref] vs Pharmacy)	0.43 (0.19,0.97)	**0.04**
**Number of days attending clinic/pharmacy**	1.03 (1.00,1.06)	**0.05**
**Aboriginal Client,** binary (non-Aboriginal [ref] vs Aboriginal)	1.85 (0.81,4.20)	0.15
**Age (years)**	1.02 (0.98, 1,06)	0.33

## Discussion

SESLHD covers nine local governments areas, expanding from Sydney Central Business District to the Royal National Park, and manages eight public hospitals, twelve community health centres, nine oral health clinics, as well as mental health, youth health, sexual health and imaging and pathology services [50]. It also offers three major public drug and alcohol clinics located in Surry Hills, Kogarah, and Sutherland [50].

Although preventative strategies such as reducing the frequency that clients are required to attend clinic/pharmacy for dosing by increasing TADs, transferring clients’ dosing points to pharmacy and encouraging clients to switch to LADB from methadone or SL-BPN have taken place nationally and globally since the outbreak of the pandemic [15, 27–31], this appears to be the first study to determine whether these strategies protected this already vulnerable population against COVID-19 infection.

Our findings demonstrate strong evidence linking employment status, vaccination status, dosing location, and frequency of clinic or pharmacy attendance, to the likelihood of attaining COVID-19 infection amongst a cohort of clients receiving ODT between August 1st, and November 30th, 2021. Clients who were required to attend clinic once a week or less for oral ODT and clients receiving LADB were both significantly less likely to acquire COVID-19 than clients required to attend for dosing six or more times a week. However, we were unable to further stratify clients into those receiving weekly versus monthly LADB due to the low sample size of the former group (*n* = 3).

Clients who identified as Aboriginal also had a higher risk of COVID-19 infection, (although not statistically significant in the multivariate logistic regression model after adjusting for other covariates). This inequity undoubtedly stems from the history of colonial oppression and the ongoing structural violence, racism, stigma, and intergenerational trauma faced by the Aboriginal community, the resultant mistrust in Australian health-care systems [51], and the consequential lower vaccination rates, poorer treatment adherence, higher rates of missed doses and higher dosing-site attendance requirements than non-Aboriginal clients. In fact, the subgroups of clients known, from prior research, to be at an inherently greater risk of COVID-19 infection (i.e. unemployed, unvaccinated and Aboriginal people) [7–10] were required, on average, to attend for dosing more frequently than their counterparts, based on risk assessments conducted by their prescribing doctors.

The significantly lower COVID-19 vaccination rates amongst our cohort (68%) vs NSW vaccination rates (83%) at the end of October 2021 [49] could be partially explained by both the younger age of our cohort (vaccinations targeted the elderly first and the AstraZeneca and Pfizer vaccines had only been implemented in NSW six months prior) [26] and that almost 20% of SESLHD ODT clients identified as Aboriginal (compared to about 3.4% of the population of NSW) [52]. This hypothesis is supported by the fact that after stratifying by age and excluding those who identified as Aboriginal, the difference in vaccination rates between the SESLHD Drug and Alcohol ODT community and those of NSW at large [49] reduced, particularly in the younger age group. As of October 21.^st^, 2021, 69% of SESLHD ODT Non-Aboriginal clients aged 16–49 were fully vaccinated compared to 70% of the NSW population aged 16–49; whilst 75% of SESLHD ODT Non-Aboriginal Clients aged 50–69 were fully vaccinated compared to 88% of the NSW population aged 50–69 [49]

Data on the proportion of Aboriginal people in NSW fully vaccinated by the end of October 2021, stratified by demographics such as age, employment status and gender, is very limited. However, it was reported that by the end of October 2021, 110,371 NSW Aboriginal people had been fully vaccinated [53], representing about 56% of the NSW Aboriginal population eligible for vaccinations at the time^3^ [52–54]. This was slightly less than the proportion of Aboriginal people attending SESLHD for ODT at the time who had been fully vaccinated -approximately 58%. This could perhaps be explained by the fact that Aboriginal people not on ODT may not regularly attend healthcare centres, and thus were less likely to be offered COVID-19 vaccinations on a regular basis.

Whilst international literature had consistently voiced that COVID-19 vaccination rates were lower amongst clients with SUDs, (e.g. in countries such as Italy [20], America [21], Canada [22] and Spain [23]), our data indicated that vaccination uptake in ODT clients was comparable to the general population after adjusting for age and whether a client identified as Aboriginal. This involved considerable effort and targeted strategies by ODT staff to increase vaccination uptake amongst clients, including providing vaccinations in ODT services, consumer worker activities and financial incentives ($20 supermarket vouchers) for clients to complete vaccination.

Finally, approximately 10% (*n* = 41) of SESLHD ODT clients contracted COVID-19 during the timeframe of the study which was over ten times the COVID acquisition rate of NSW adults at large across the same time span (0.8%, *n* = 48,558) [34, 35]. In addition to their requirements to regularly attend clinics/pharmacies for dosing, this also could be explained by their higher rates of unemployment (73% vs 4%) [55], homelessness (5% vs 0.5%) [56] and Aboriginality (18% vs 3.4%) [52], as well as their lower vaccination rates (68% vs 83% as of October 21st, 2021) [49] and substance use.

This study is relevant as it is the first to quantify the effectiveness, in terms of COVID-19 acquisition, of relaxing requirements for ODT clients to dose at pharmacy and of reducing the frequency of attendance required for supervised dosing. Had these measures not been implemented, our findings suggest that SESLHD ODT clients would likely have suffered significantly higher rates of COVID-19 acquisition with potentially serious complications. However, this study also revealed that clients whose demographics had already placed them at a higher risk of COVID-19 infection (e.g. unemployed, homeless, Aboriginal people) were less likely to have received these treatment adjustments. Much of this inequity can be explained by the structural violence, stigma and/or discrimination faced by these groups resulting in a reluctance to access and trust healthcare services and providers [51], Although much progress has been made in terms of the prevention (e.g. ongoing vaccine developments and roll-outs) and pharmacological management of COVID-19, it is generally agreed that the COVID-19 pandemic is still far from over [57]. Thus, further research into how to safely and effectively reduce the frequency of clinic or pharmacy attendance required by this subgroup of clients should remain a priority.

The results of this study are limited as these measures were established across SESLHD ODT clinics at the beginning of the first wave of COVID-19 in NSW (April 2020), prior to any recorded cases occurring amongst SESLHD ODT clients, meaning whilst we were able to conclude that clients who received these interventions were less likely to acquire COVID-19 than those who did not, we could not definitively establish that these interventions themselves caused a reduction in COVID-19 infection rates amongst clients.

## Conclusion

There is a clear association between dosing site, required attendance and vaccination status of clients, and COVID-19 acquisition, highlighting the importance of adjustments to the ODT model of care in preventing COVID-19 transmission, not only to clients, but also to healthcare workers in these settings. The fact that the more vulnerable clients already at a higher risk of COVID-19 (e.g. clients identifying as Aboriginal and unemployed clients) were required to attend more frequently for dosing and were less likely to be vaccinated calls for further research targeting these subgroups to appropriately address these modifiable risk factors.

## Data Availability

The datasets used and/or analysed during the current study are available from the corresponding author on reasonable request.

## Abbreviations

COVID-19: Coronavirus-19
ODT: Opioid dependency treatment
SESLHD: South Eastern Sydney local health district
TAD: Takeaway doses
LADB: Long-acting depot buprenorphine
eMR: Electronic medical records
AIR: Australian immunisation register
NSW: New South Wales
SUD: Substance use disorder
SL-BPN: Sublingual buprenorphine
PPE: Personal protective equipment
PCR: Polymerase chain reaction
RAT: Rapid antigen testing
NCIMS: Notifiable conditions information management system
ATOP: Australian treatment outcomes profile
PHU: Public health unit
MSE: Mean squared error

## Footnotes

1 SESLHD HREC is constituted and operates in accordance with the National Health and Medical Research Council’s (NHMRC) *National Statement on Ethical Conduction in Human Research (2007),* NHRMC and Universities Australia *Australian Code for the Responsible Conduct of Research (2007)* and the *CPMP/ICH Note for Guidance on Good Clinical Practice.*

2 Henceforth the term “Aboriginal” is used to represent “Aboriginal and Torres Strait Islander”, in recognition that Aboriginal people are the original inhabitants of New South Wales.

3 It is estimated that in 2021 approximately 292,147 Aboriginal Australians lived in NSW of which 68% were > 15 years old.
